# When Foreign Body Aspiration Is Not the Whole Story: An Undiagnosed Congenital Pulmonary Airway Malformation Mimicking Tension Pneumothorax in an Infant

**DOI:** 10.1002/ccr3.72560

**Published:** 2026-04-17

**Authors:** Husam Ibrahımoglu, Zeynep S. K. Demir, Elif N. Ayaz, Ayla Gunkizil, Serkan Ozsoylu, Ayse N. Deger, Ibrahim Uygun

**Affiliations:** ^1^ Department of Pediatric Surgery Kutahya City Hospital, Kutahya University of Health Sciences Kutahya Türkiye; ^2^ Department of Pediatrics, Pediatric Intensive Care Unit Kutahya City Hospital, Kutahya University of Health Sciences Kutahya Türkiye; ^3^ Department of Pathology Kutahya City Hospital, Kutahya University of Health Sciences Kutahya Türkiye

**Keywords:** congenital pulmonary airway malformations, diagnostic dilemma, foreign body aspiration, hypoxic–ischemic brain injury, pediatric emergency surgery

## Abstract

Failure to improve after initial management in infants with suspected foreign body aspiration and apparent tension pneumothorax should prompt reconsideration of the diagnosis, including underlying congenital lung anomalies such as CPAM.

## 1 Introduction

Congenital pulmonary airway malformations (CPAM) are cystic lesions of the lung parenchyma that maintain communication with the tracheobronchial tree and represent the most common congenital pulmonary malformations in children [1]. The clinical presentation of CPAM is highly variable, ranging from severe neonatal respiratory distress to completely asymptomatic cases that may remain undetected for prolonged periods [2, 3]. In asymptomatic patients, effective compensatory ventilation of the contralateral lung may mask significant functional impairment, resulting in a critically reduced pulmonary reserve despite normal growth and development [1, 4]. Foreign body (FB) aspiration is a major cause of acute respiratory compromise, morbidity, and mortality in infants and young children and may rapidly progress to severe hypoxia if not promptly recognized and treated [5]. In emergency settings, initial management is guided by life‐threatening clinical and radiological findings, often necessitating immediate airway intervention and resuscitative measures before definitive diagnostic clarification can be achieved. The coexistence of FB aspiration and an undiagnosed CPAM represents a diagnostic and therapeutic challenge. While FB aspiration is typically the primary precipitating event, an underlying congenital lung malformation may significantly exacerbate the severity of hypoxia by limiting pulmonary reserve and complicating radiological interpretation [1, 4]. In such situations, CPAM may mimic conditions such as tension pneumothorax on plain chest radiography, particularly following positive‐pressure ventilation, and advanced imaging may be required when initial interventions fail to produce the expected clinical improvement [6, 7, 8, 9].

Here, we present a pediatric case in which FB aspiration led to severe hypoxic–ischemic brain injury in an infant with a previously asymptomatic and undiagnosed CPAM.

## 2 Case History/Examination

An 11‐month‐old male infant with no significant past medical history, was brought to a hospital in another city with suspected FB aspiration. Antenatal history was unremarkable, with no parental consanguinity or known chronic diseases in the family. Review of national health records and detailed family interviews confirmed normal growth and age‐appropriate development, with no history of respiratory symptoms, recurrent infections, prior hospital admissions, or previous lung imaging.

The event occurred when the mother was preparing peanuts for the infant's 4‐year‐old sibling and briefly left the room. Shortly thereafter, she heard the infant coughing and returned to find him in severe respiratory distress. An attempt at the Heimlich maneuver was unsuccessful. The sibling later admitted to placing peanuts into the infant's mouth. The grandfather was called for assistance and found the infant unconscious upon arrival, prompting immediate transport to the emergency department.

**FIGURE 1:**
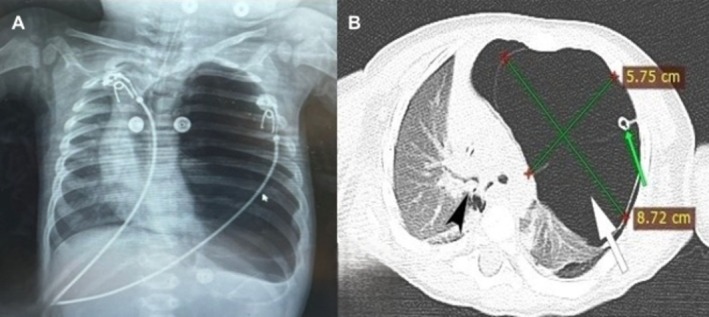
(A) Chest radiograph obtained at the referring hospital demonstrating marked hyperlucency of the left hemithorax with rightward mediastinal shift, initially interpreted as left‐sided tension pneumothorax. (B) Axial computed tomography (CT) image of the chest showing a large multicystic lesion occupying the left hemithorax consistent with congenital pulmonary airway malformation (white arrow), a foreign body obstructing the right main bronchus with extension into the middle and lower lobar bronchi (black arrow), and a left‐sided thoracic tube (green arrow).

On arrival at the emergency department, the infant was pulseless. Cardiopulmonary resuscitation was initiated immediately and return of spontaneous circulation was achieved after 15 min. The patient was intubated during resuscitation and ventilated with positive pressure. Initial chest radiography demonstrated marked hyperlucency of the left hemithorax with rightward mediastinal shift, raising strong suspicion for left‐sided tension pneumothorax (Figure 1A).

Given the acute clinical context of FB aspiration and cardiopulmonary arrest, the radiographic findings were initially interpreted as tension pneumothorax secondary to airway obstruction caused by the aspirated foreign body. Accordingly, an emergent left‐sided tube thoracostomy was performed with the primary aim of relieving the mediastinal shift and restoring hemodynamic stability. However, no significant clinical improvement was observed following chest tube placement, and the patient was therefore referred to our institution and subsequently admitted to the pediatric intensive care unit for further diagnostic evaluation.

On admission to the pediatric intensive care unit, the patient was intubated and mechanically ventilated. Neurological examination revealed unconsciousness, flexor response to pain, hyperreflexia, and bilateral clonus. Heart sounds were more prominent over the right hemithorax, and vital signs were stable for age.

Arterial blood gas analysis revealed severe metabolic acidosis with elevated lactate levels. The patient underwent reintubation, and a right subclavian central venous catheter was placed.

## 3 Differential Diagnosis, Diagnostic Investigation, and Treatment

In the acute setting, the leading diagnosis was tension pneumothorax secondary to foreign body aspiration, based on sudden cardiopulmonary collapse, mediastinal shift on chest radiography, and hemodynamic instability. Massive atelectasis and obstructive emphysema were also considered. Because no clinical improvement occurred after tube thoracostomy, the chest tube system was revised; however, absent left‐sided breath sounds and lack of oscillation raised suspicion for additional pathology. Thoracic CT was therefore obtained without delay and demonstrated a large multicystic lesion in the left hemithorax consistent with CPAM, together with a foreign body in the right main bronchus (Figure 1B). These findings suggested that the apparent tension pneumothorax reflected CPAM‐related air trapping under positive‐pressure ventilation rather than true pneumothorax.

**FIGURE 2:**
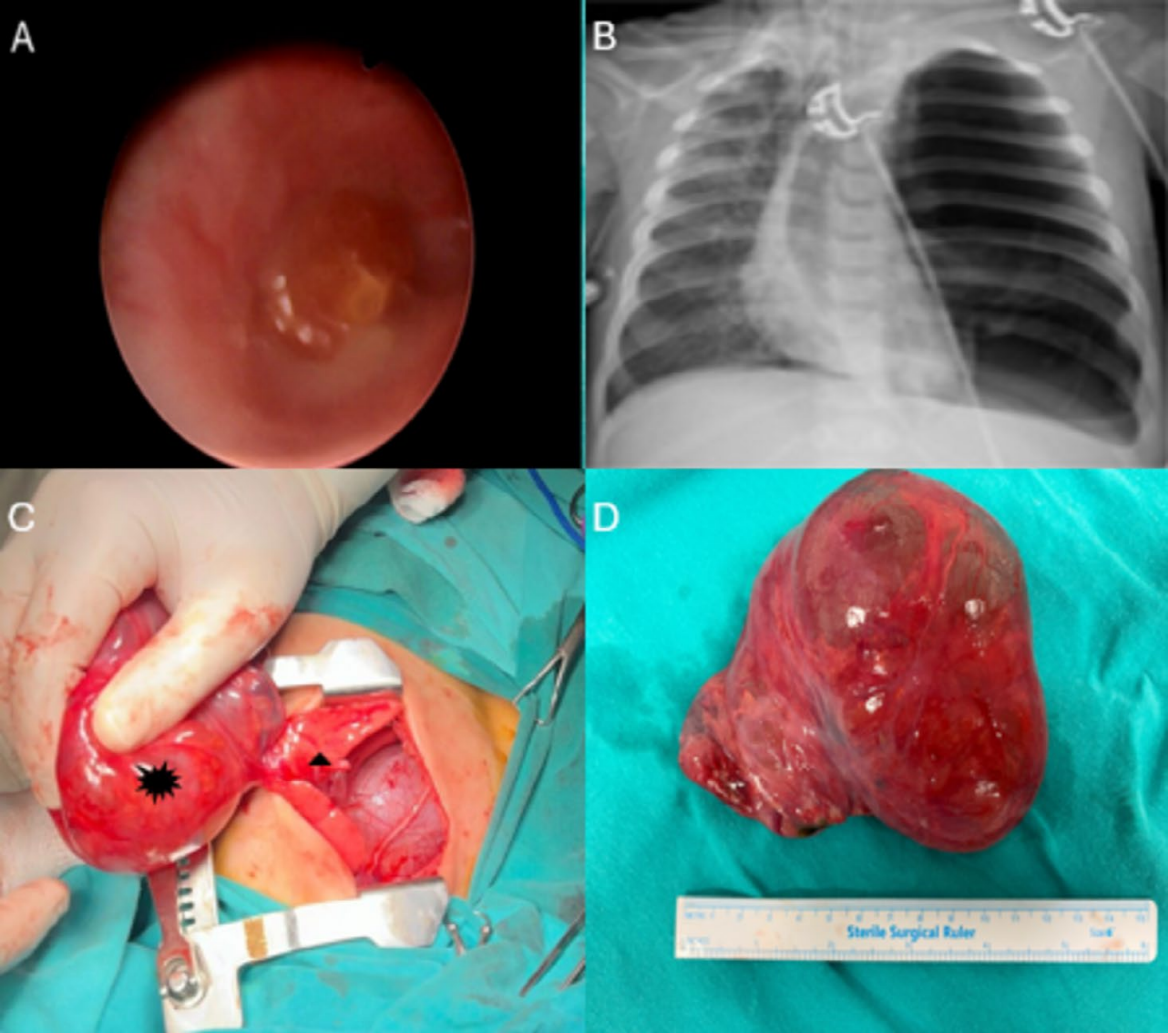
(A) Bronchoscopic view demonstrating a foreign body lodged in the right main bronchus, configured in a manner that left the right upper lobe bronchus patent while completely obstructing the middle and lower lobar bronchi. (B) Postbronchoscopy chest radiograph obtained in the absence of a chest tube, demonstrating reduced left‐sided hyperinflation with partial aeration of the left lower lobe, consistent with resolution of airway‐related air trapping rather than persistent pneumothorax. (C) Intraoperative view showing a large cystic lesion arising from the upper lobe of the left lung and occupying the left hemithorax (black asterisk), with compression and atelectasis of the adjacent left lung parenchyma (black arrowhead). (D) Gross specimen of the excised cystic lesion.

The patient then underwent urgent bronchoscopy, which revealed and allowed removal of a peanut from the right main bronchus (Figure 2A). Because the chest tube had not achieved effective decompression and was considered potentially unhelpful, it was removed during the procedure. Subsequent chest radiography showed reduced left‐sided hyperinflation and partial re‐aeration of the left lower lobe, supporting resolution of airway‐related air trapping rather than persistent pneumothorax (Figure 2B). Owing to critical neurological and respiratory instability, definitive resection was deferred.

**FIGURE 3:**
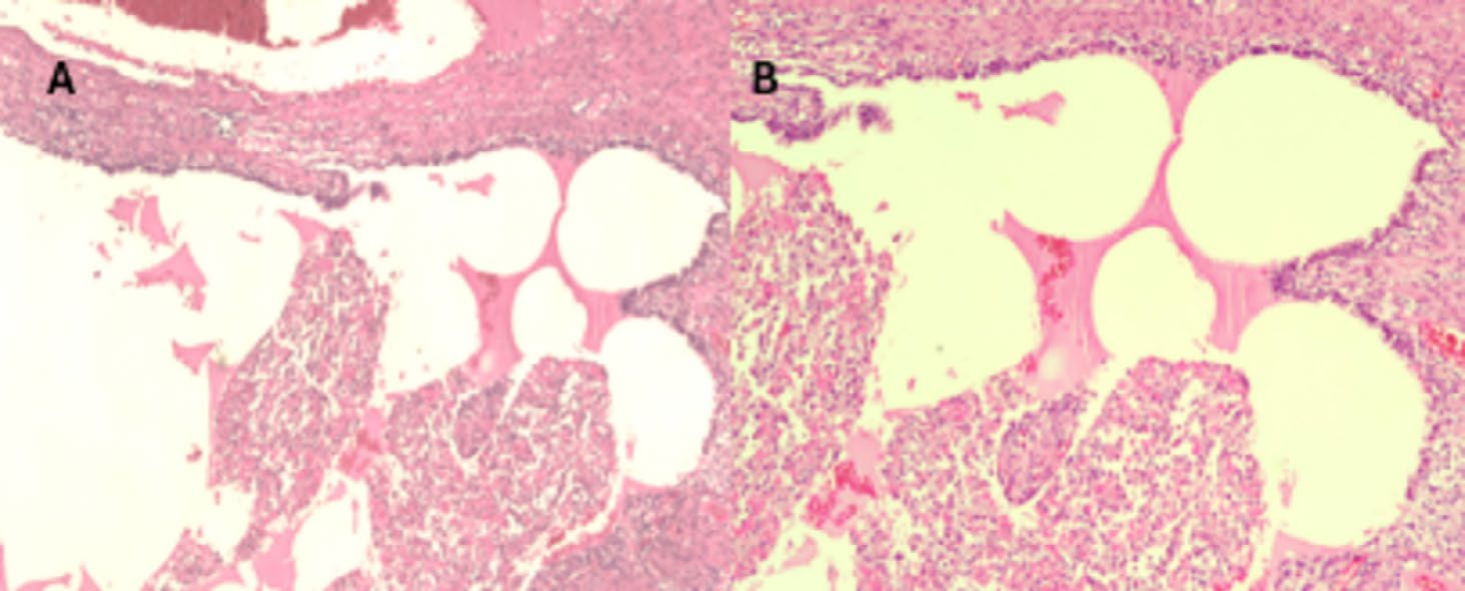
Histopathological examination of the resected lesion. (A) Hematoxylin and eosin stain (×40) showing cystic structures lined by stratified cuboidal epithelium, consistent with type 2 congenital pulmonary airway malformation. (B) Hematoxylin and eosin stain (×100) demonstrating areas lined by single‐layered cuboidal epithelium.

During follow‐up, the patient developed refractory status epilepticus, and brain magnetic resonance imaging on hospital day 6 demonstrated widespread cortical edema consistent with hypoxic–ischemic encephalopathy. After stabilization, definitive surgery was performed on hospital day 8 through a left posterolateral thoracotomy. A large cystic lesion arising from the left upper lobe was identified and excised by segmentectomy (Figure 2C,D). Histopathological examination confirmed type 2 CPAM (Figure 3A,B).

## 4 Outcome and Follow‐Up

Despite initial respiratory stabilization, the patient developed severe neurological sequelae, including lower extremity spasticity and contractures. Because of persistent neurological impairment with inadequate airway protection and absent swallowing reflex, tracheostomy and percutaneous endoscopic gastrostomy were performed during follow‐up. However, the patient died of an infectious complication during follow‐up.

## 5 Discussion

Congenital pulmonary airway malformation (CPAM) is the most common congenital lung anomaly, with an estimated prevalence of 1 in 25,000–35,000 live births, although the true incidence is likely underestimated due to the high proportion of asymptomatic cases [1]. The pathogenesis of CPAM remains incompletely understood and has been attributed to abnormalities in lung organogenesis, including dysregulated cellular proliferation, apoptosis, and aberrant branching morphogenesis at various stages of tracheobronchial development [10, 11, 12]. This developmental heterogeneity accounts for the wide spectrum of clinical presentations observed in CPAM.

The Stocker classification system, initially proposed in 1977 and later expanded in 2009, remains the most widely used framework for categorizing CPAM into five histopathological subtypes [13, 14]. Type 2 CPAM accounts for approximately 20% of cases and is frequently associated with congenital anomalies involving the gastrointestinal, genitourinary, cardiovascular, and central nervous systems [15, 16, 17]. Nevertheless, CPAM—particularly in the absence of associated anomalies—may remain clinically silent for prolonged periods, allowing normal growth and development despite a significantly reduced functional pulmonary reserve.

Prenatal ultrasonography enables detection of CPAM in the majority of cases during the second trimester, with fetal magnetic resonance imaging serving as a valuable adjunct for anatomical characterization and risk stratification [18]. Postnatally, CPAM may present with respiratory distress, recurrent infections, or air trapping; however, a substantial proportion of cases remain clinically silent and may go undetected into infancy or childhood [19, 20, 21, 22]. Reported rates of asymptomatic presentation range from 25% to 60%, contributing to delayed diagnosis and reduced clinical suspicion [4]. Importantly, CPAM may both cause spontaneous pneumothorax and radiographically mimic acute thoracic emergencies, particularly in the setting of positive‐pressure ventilation [20, 21]. While plain chest radiography raises suspicion for CPAM in only 50%–60% of cases, computed tomography provides near‐complete diagnostic accuracy, with definitive confirmation achieved through histopathological examination [18].

The optimal management of CPAM remains controversial. Although conservative follow‐up may be appropriate in carefully selected asymptomatic patients, many authors advocate for elective surgical resection to prevent complications such as recurrent infection, pneumothorax, and malignant transformation [23, 24, 25]. When respiratory compromise occurs, management should be individualized, prioritizing adequate ventilatory support and appropriately timed surgical intervention. Careful surgical timing following stabilization is critical for restoring pulmonary function and minimizing perioperative morbidity [26].

In the present case, CPAM was not the primary cause of the acute event but rather a critical aggravating factor that markedly reduced pulmonary reserve. The life‐threatening deterioration was precipitated by foreign body aspiration, while the previously undiagnosed CPAM amplified the severity of hypoxia by limiting compensatory ventilation. Accordingly, definitive surgical resection was appropriately deferred until neurological and respiratory stabilization was achieved, allowing initial management to focus on reversing the primary, reversible cause of collapse.

Foreign body aspiration remains a leading cause of acute respiratory compromise and mortality in young children, particularly those under three years of age [27, 28, 29]. Acute airway obstruction may rapidly progress to hypoxia, cardiac arrest, and hypoxic–ischemic brain injury if not promptly recognized. Although chest radiography may be inconclusive, bronchoscopy remains the gold standard for both diagnosis and treatment [30, 31]. In acute pediatric airway emergencies, an initial management algorithm prioritizing rapid physiological stabilization—most commonly tube thoracostomy for suspected tension pneumothorax followed by bronchoscopic airway evaluation—is a reasonable approach in selected emergency settings [32].

In this patient, bronchoscopic airway clearance resulted in hemodynamic stabilization, allowing major thoracic surgery to be deferred. Because the clinical situation was no longer emergent after stabilization, definitive surgical intervention was not undertaken during nighttime hours. In such settings, postponing major surgery to daytime hours is widely considered a safer approach, allowing optimal operative conditions, full multidisciplinary team availability, and reduced perioperative risk, particularly in pediatric patients [33, 34].

This case highlights that, in life‐threatening pediatric airway emergencies, failure to achieve the expected clinical response after initial stabilization measures should prompt timely escalation to advanced imaging. In cases where clinical findings and response to initial management are incongruent, early use of low‐dose CT may facilitate timely identification of both airway obstruction and underlying congenital anomalies [18, 30, 31]. Early recognition of diagnostic complexity and appropriate sequencing of airway management, imaging, and definitive surgery were key to the successful management of this challenging scenario.

## 6 Conclusion

This case highlights how a clinically silent congenital pulmonary airway malformation may complicate an acute airway emergency by reducing pulmonary reserve. In infants presenting with cardiopulmonary collapse after foreign body aspiration, initial management should focus on physiological stabilization. Lack of improvement after appropriate chest decompression should prompt reconsideration of the diagnosis and further imaging. In the present case, timely computed tomography, bronchoscopic airway stabilization, and delayed definitive surgery after clinical stabilization were key components of management.

## Author Contributions

**Husam Ibrahımoglu:** conceptualization, data curation, investigation, writing – original draft, writing – review and editing. **Zeynep S. K. Demir:** data curation, investigation, writing – review and editing. **Serkan Ozsoylu:** supervision, validation, writing – review and editing. **Ayla Gunkizil:** data curation, investigation, writing – review and editing. **Ayse N. Deger:** data curation, validation, writing – review and editing. **Ibrahim Uygun:** conceptualization, supervision, validation, writing – review and editing. **Elif N. Ayaz:** data curation, investigation, writing – review and editing.

## Funding

The authors have nothing to report.

## Disclosure

Authorship: All authors attest that they meet the current ICMJE criteria for authorship.

## Consent

Written informed consent was obtained from the patient's legal guardians for publication of this case report and any accompanying images.

## Conflicts of Interest

The authors declare no conflicts of interest.

## Data Availability

The data supporting the findings of this case report are available from the corresponding author upon reasonable request. Due to the nature of this study and patient confidentiality considerations, the data are not publicly available.
